# The Roles of Circadian Clock Genes in Plant Temperature Stress Responses

**DOI:** 10.3390/ijms25020918

**Published:** 2024-01-11

**Authors:** Juna Jang, Sora Lee, Jeong-Il Kim, Sichul Lee, Jin A. Kim

**Affiliations:** 1 Department of Agricultural Biotechnology, National Academy of Agricultural Science, Rural Development Administration, Jeonju 54874, Republic of Korea; jangjuna99@korea.kr (J.J.); sora19@korea.kr (S.L.); scironlee@gmail.com (S.L.); 2 Department of Integrative Food, Bioscience and Biotechnology, Chonnam National University, Gwangju 61186, Republic of Korea; kimji@chonnam.ac.kr

**Keywords:** circadian clock genes, temperature response, *DREB1*, *PIF4*, *TOC1*

## Abstract

Plants monitor day length and memorize changes in temperature signals throughout the day, creating circadian rhythms that support the timely control of physiological and metabolic processes. The *DEHYDRATION-RESPONSE ELEMENT-BINDING PROTEIN 1/C-REPEAT BINDING FACTOR* (*DREB1/CBF*) transcription factors are known as master regulators for the acquisition of cold stress tolerance, whereas *PHYTOCHROME INTERACTING FACTOR 4* (*PIF4*) is involved in plant adaptation to heat stress through thermomorphogenesis. Recent studies have shown that circadian clock genes control plant responses to temperature. Temperature-responsive transcriptomes show a diurnal cycle and peak expression levels at specific times of throughout the day. Circadian clock genes play essential roles in allowing plants to maintain homeostasis by accommodating temperature changes within the normal temperature range or by altering protein properties and morphogenesis at the cellular level for plant survival and growth under temperature stress conditions. Recent studies revealed that the central oscillator genes *CIRCADIAN CLOCK ASSOCIATED 1*/*LATE ELONGATED HYPOCOTYL* (*CCA1/LHY*) and *PSEUDO-RESPONSE REGULATOR5*/*7*/*9* (*PRR5*/*7*/*9*), as well as the *EVENING COMPLEX* (*EC*) genes *REVEILLE4/REVEILLE8* (*REV4*/*REV8*), were involved in the *DREB1* pathway of the cold signaling transcription factor and regulated the thermomorphogenesis gene *PIF4*. Further studies showed that another central oscillator, *TIMING OF CAB EXPRESSION 1* (*TOC1*), and the regulatory protein *ZEITLUPE* (*ZTL*) are also involved. These studies led to attempts to utilize circadian clock genes for the acquisition of temperature-stress resistance in crops. In this review, we highlight circadian rhythm regulation and the clock genes involved in plant responses to temperature changes, as well as strategies for plant survival in a rapidly changing global climate.

## 1. Introduction

Every organism living on the rotating Earth has evolved a circadian clock that regulates biological processes on the basis of environmental signals, which change according to the time of day [1]. The circadian clock is a molecular timing device that generates the physiological and biological rhythms of numerous developmental processes in 24 h cycles in most living organisms [2]. Circadian rhythms are endogenously generated and self-maintaining, allowing them to preserve robust and precise timing across a wide range of physiological temperatures [3,4]. The clock is a significant regulator of plant life, linked to various signaling and metabolic pathways that promote development and environmental adaptation responses [5,6,7]. In many plant species, the correct synchronization of the clock with the environment contributes to fitness, ensuring survival in addition to optimal growth and performance under fluctuating environmental conditions.

Changes in temperature throughout the day act as entrainment signals that provide time information to plants, supporting the creation of a rhythm [8]. Temperature also affects crop quality and productivity, plant growth and development, and geographic distribution. Plants continually adapt to seasonal changes in temperature, optimizing their growth and development. Temperature-dependent plant responses include thermoperiodism, thermomorphogenesis, cold stratification, and extreme-temperature responses. Recent studies have revealed mechanisms that stabilize the clocks and circadian rhythms even under extreme temperature changes or stressful conditions that affect plant growth and development. Representative examples show that plants have unique mechanisms for various temperature conditions: the alternative splicing of the *CIRCADIAN CLOCK ASSOCIATED 1*(*CCA1*) gene occurs under low-temperature conditions [9], whereas E3 ubiquitin ligase *ZEITLUPE* (*ZTL*) and *HEAT SHOCK PROTEIN 90* (*HSP90*) stabilize the biological clock under high-temperature conditions [10]. Because environmental changes affect plant growth and development, research concerning mechanisms of adaptation to temperature changes is critical for efforts to understand the abilities of plants to adapt to temperature stresses and maintain productivity.

## 2. The Plant Circadian Clock

### 2.1. Circadian Feedback Loops in Plants and Environmental-Stress-Related Clock Genes

Plant clock systems are complex genetic circuits containing multiple inhibitory feedback loops [11]. The transcriptional regulatory network model system that regulates circadian rhythm has been widely studied in *Arabidopsis thaliana*. The feedback loop of the main oscillator consists of *CCA1*, *LATE ELONGATED HYPOCOTYL* (*LHY*), *TIMING OF CAB EXPRESSION 1* (*TOC1*) (also known as *PSEUDO-RESPONSE REGULATOR1, PRR1*), the MYB transcription factor *LUX ARRHYTHMO (LUX)*, and *EVENING COMPLEX* (*EC*), as well as the transcriptional regulatory factors *EARLY FLOWERING 3 (ELF3)* and *ELF4*. In this central oscillator feedback loop, *CCA1* and *LHY* show peak expressions in the morning and repress the transcription of *TOC1*; in turn, TOC1 regulates *CCA1* and *LHY* expression in the late evening. Additionally, *CCA1* and *LHY* activate the *PRR7* and *PRR9* genes, which then repress *CCA1* gene transcription in a temporal sequence. *TOC1* represses the transcription of *EC* components, *PRRs*, and *GIGANTEA* (*GI*). Other major components of the central oscillator include *GI*, *ZTL*, and *PRR3*. *LHY* and *CCA1* repress *GI* transcription in the early morning, whereas *GI* transcription peaks in the middle of the day [12,13].

Mutations in circadian-rhythm-regulating clock genes affect the function of the central oscillator, thereby modifying the period and amplitude of the rhythm, while altering the plant response and adaptive capacity to various environmental stress conditions. The *prr5/7/9* triple mutant is more tolerant to high salinity and drought stress [14]. *GI*-overexpressing plants show enhanced salt sensitivity, whereas *gi* mutants show enhanced salt tolerance and improved survival under drought and oxidative stress [15,16]. The reduced expression of *TOC1* in *TOC1*-RNAi plants improves drought tolerance, whereas the overexpression of *TOC1* increases water loss and reduces survival under drought conditions [17]. *prr5/7/9* and *toc1* mutants show significantly increased freezing tolerance [14,18]. In contrast, *gi*, *lux*, *lhy*, *cca1*, and *lhy*×*cca1* mutants exhibit decreased freezing tolerance [19,20,21,22]. In addition to mechanistic studies in model plants, analyses of many crops have been conducted [23,24,25,26,27,28,29,30,31,32,33,34,35,36,37] (Table 1). These findings indicate that the circadian clock contributes to the ability of plants to tolerate environmental stresses.

### 2.2. Clock Genes Related to Temperature Compensation Mechanisms

The operation and maintenance of biological systems with precise responses to environmental changes is essential for plant survival and optimal growth. Plant circadian clocks have a molecular buffering system that allows for the maintenance of robust circadian rhythms, rendering the clock output pathway insensitive to physiological ranges of temperature fluctuations; this phenomenon is known as temperature compensation [38]. Temperature compensation mechanisms are highly conserved and considered essential properties of circadian clocks within living organisms. Studies with model plants indicated that a group of central clock components is involved in temperature compensation processes [39,40]. Arabidopsis plants lacking *PRR7* and *PRR9* could not maintain circadian rhythms in response to warm–cold cycles, suggesting that PRR proteins are crucial for the temperature entrainment of the clock [41]. *GI* is also important for the *CCA1/LHY*-mediated buffering of robust circadian rhythms; it serves as a potential regulator of temperature compensation [40]. Temperature compensation mechanisms are highly conserved and considered essential properties of circadian clocks within living organisms [26,42,43].

Studies to date have shown that the ZTL and HSP protein groups, as well as the FLOWERING BASIC HELIX-LOOP-HELIX 1 (FBH1) and HEAT SHOCK FACTOR (HSF) protein groups, are crucial components of the plant circadian clock response to warm temperatures [10]. The overproduction of FBH1 alters the clock period by regulating the *CCA1* response to temperature changes [44]. The heat-responsive *HsfB2b* transcription factor also inhibits *PRR7* transcription by binding to the promoter under elevated temperature conditions [45]. In contrast, a lack of *HsfB2b* causes the circadian period to be shortened under the same temperature conditions. These findings suggest that circadian clock genes transmit temperature signals and may affect resistance to temperature stress through mechanisms similar to other abiotic stress responses. Although it is unclear whether clock components operate independently or cooperatively through an integrated signaling network during the temperature compensation process, the relationships among clock genes and temperature response genes have attracted the attention of scientists interested in establishing temperature stress resistance in plants through the use of clock genes.

## 3. Circadian Clock Gene Responses under Cold Stress

### 3.1. Cold Stress Response in Plants

In Arabidopsis, the *DEHYDRATION-RESPONSE ELEMENT-BINDING PROTEIN 1/C-REPEAT BINDING FACTORs* (*DREB1*/*CBFs*) were identified as major transcription factors involved in cold-responsive gene expression [46,47]. These *DREB1*/*CBFs* are *APETALA2*/*ETHYLENE-RESPONSIVE FACTOR* (*AP2*/*ERF*) transcription factors, which bind to *DEHYDRATION-RESPONSE ELEMENT* (*DRE*) and act as master regulators in cold-induced gene expression. There are three *DREB1*/*CBF* genes in Arabidopsis: *DREB1A*/*CBF3*, *DREB1B*/*CBF1*, and *DREB1C*/*CBF2*. These genes are arranged adjacent to each other in the Arabidopsis genome in the following order: *DREB1B*, *DREB1A*, and *DREB1C*. Arabidopsis mutant plants with all three genes disrupted exhibit severely impaired freezing tolerance. All three genes are rapidly induced by cold stress, and the induction of the *DREB1* gene triggers a cold-responsive transcription cascade, leading to the expression of multiple cold-inducible genes. Therefore, research concerning the *DREB1* expression mechanism in response to cold stress is important for understanding plant perception and response. Members of the CALMODULIN-BINDING TRANSCRIPTION ACTIVATOR (CAMTA) protein family are transcriptional activators that trigger the expression of *CBF* and positively regulate freezing tolerance in Arabidopsis. *CAMTA3* and *CAMTA5* regulate *CBF1* and *CBF2* expression in response to rapid temperature decreases but not in response to gradually decreasing temperatures.

### 3.2. Cold Signaling Pathways Involving Clock-Related Transcription Factors

Many cold-induced genes, including *DREB1A*, are expressed at low levels around noon under normal temperature conditions [48,49]. Mutations in genes encoding *PRR9*, *PRR7*, and *PRR5*—the central circadian oscillator—enhanced the expression of the *DREB1* gene and its downstream genes [14,50]. Additionally, the cold-induced expression of the *DREB1* gene was significantly reduced in double-mutant plants of *CCA1* and *LHY*. These results indicate that many cold-induced genes, including *DREB1A*, are regulated by clock genes and thus likely to affect the plant response to low temperature.

**Figure 1:**
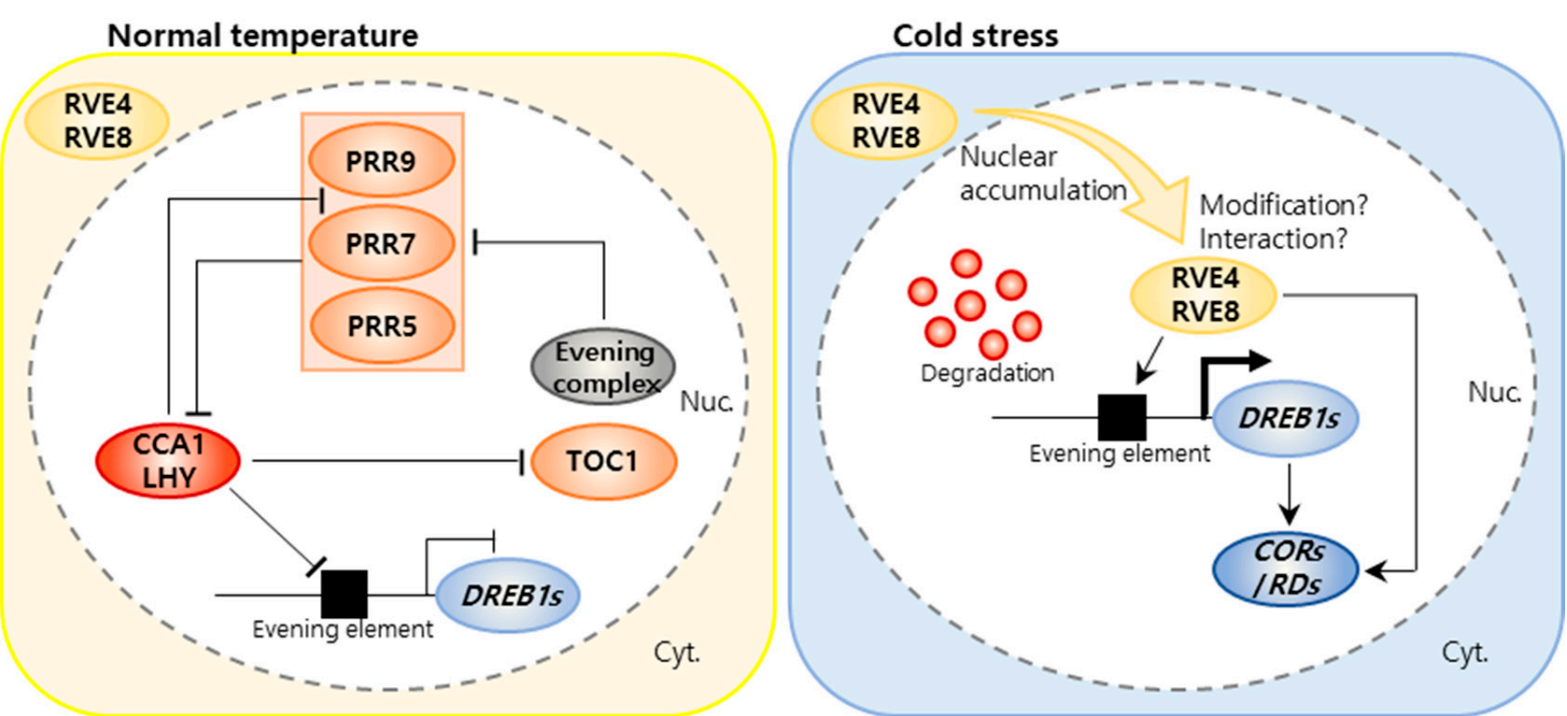
Schematic model for the expression of *DREB1* genes under normal growth (22° C) and cold stress (4 °C) conditions. Under normal growth conditions, CIRCADIAN CLOCK ASSOCIATED 1 (CCA1), LATE ELONGATED HYPOCOTYL (LHY), and PSEUDO-RESPONSE REGULATORs (PRRs) suppress the expression of *DEHYDRATION-RESPONSE ELEMENT-BINDING PROTEIN 1* (*DREB1*). Under cold stress conditions, REVEILLE4 (RVE4)/REVEILLE8 (RVE8) accumulate in nuclei, and CCA1 and LHY are degraded. RVE4 and RVE8 induce the expression of *DREB1* genes through the cis-acting evening element. RVA4/RVE8 also directly regulate the expression of *COLD-REGULATED/RESPONSIVE TO DEHYDRATION* (*COR/RD*) genes [55].

In addition to *CCA1* and *LHY*, regulatory factors include *REVEILLE4*/*LHY-CCA1-LIKE1* (*RVE4*/*LCL1*) and *RVE8*/*LCL5*, which regulate *DREB1* expression by binding to the *EVENING ELEMENT* (*EE*) in the *DREB1* promoter region (Figure 1). *EE* directly binds to promoters in the circadian clock and activates the expression of evening genes repressed by *CCA1* and *LHY* [51,52,53]. Under low-temperature conditions, RVE4 and RVE8 bind directly to the *DREB1* promoter and function as transcriptional activators. The function of RVE6 overlaps with the functions of RVE3 and RVE5, but RVE6 conditionally activates *DREB1* expression only when the activities of RVE4 and RVE8 are lost. Additionally, the RVE4 and RVE8 proteins are rapidly and reversibly transported from the cytoplasm to the nucleus in response to cold stress, thus activating the expression of *DREB1*. Under normal stress-free conditions, CCA1 and LHY proteins bind to the *DREB1* promoter and act as transcriptional repressors of *DREB1* expression. In particular, they are rapidly degraded under cold stress conditions to induce *DREB1* expression. However, some CCA1 and LHY proteins are not degraded; they exist at low levels under cold stress conditions and bind to RVE1 and RVE2 to suppress the expression of the cold-induced gene *DREB1* [54]. CCA1 and LHY bind to RVE4/RVE8 under cold stress conditions and indirectly increase the expression of *DREB1* while directly regulating the expression of *COLD-REGULATED/RESPONSIVE TO DEHYDRATION* (*COR*/*RD*) genes without the mediation of DREB1/CBFs (Figure 1). These findings indicate that clock-related transcription factors play an essential role in triggering the expression of cold-induced genes. Studies on their mechanisms will reveal the complex relationships of plant biological clocks with cold stress responses, resulting in breeding materials that can be used to develop cold-stress-resistant crops.

### 3.3. Clock Genes Involved in Detecting Temperature Decreases

EC, composed of the DNA-binding proteins LUX, ELF3, and ELF4, is a transcriptional repression complex that constitutes a crucial component of the plant circadian clock. EC regulates temperature-dependent plant growth in the normal temperature range. EC can act as a temperature sensor through its stronger DNA binding at 4 °C and weaker binding at 27 °C; these binding interactions are regulated by ELF4. Moreover, ELF4 can move from shoots to roots; low temperature enhances this movement [56]. ELF3 is a scaffold protein and vital component of temperature-sensing machinery. Polyglutamine repeats in the prion-like domain of ELF3 are required for its function as a temperature sensor [57]. However, low-temperature detection by ELF3 below 12° C has not yet been demonstrated. The post-translational regulation of clock-related MYB, such as the nuclear accumulation of RVE4 and RVE8 and the degradation of CCA1 and LHY, occurs in response to cold stress. Detailed mechanistic analyses of the cold-responsive regulation of circadian components may lead to the elucidation of temperature-sensing systems.

## 4. Circadian Clock Genes Associated with Heat Stress Responses

### 4.1. Responsive Transcriptomes Revealing a Diurnal Response to Heat Stress in Plants

In recent decades, studies have demonstrated that circadian clock genes are also involved in plant responses to heat stress [58,59]. Although the relationship between the low-temperature stress response and the circadian clock has been widely documented, the circadian clock contribution to the heat stress response remains largely unknown. Research concerning plant clock genes involved in heat stress adaptation is ongoing [60,61,62,63].

Based on transcriptome experiments, up to 50% of the genes responsive to heat, cold, salinity, osmotic pressure, or water deprivation exhibit circadian rhythmicity in Arabidopsis [17,64,65]. Heat stress also significantly perturbs the transcriptome, ~70% of which displays a time-of-day (i.e., ZT1 or ZT6) response [66]. Enriched gene ontology functional categories related to high-temperature stress include many of the known heat shock transcription factors (HSFs) and heat shock proteins (HSPs) [67]. Whereas the differentially expressed genes (DEGs) of HSFs and HSPs are primarily upregulated in response to heat stress, DEGs of categories including response to abscisic acid, alcohol, and lipids are downregulated in response to heat stress conditions. Experimental analyses showed that these DEGs were enriched at the time points ZT1 and ZT6. For example, the induction of *HSFA3* in response to heat stress was greater at ZT6 relative to the wild type, suggesting that *HSFA3* is more sensitive to heat stress in the early afternoon. Intriguingly, the induction of *HSFA3* was strongly enhanced in *cca1lhy* and *prr7prr9* mutants at 37 °C relative to 22 °C, suggesting that the clock also plays an important role in modulating the transcriptional response of *HSFA3* to high-temperature stress.

Additionally, *CCA1*, *PRR7*, and *PRR9* were upregulated under high-temperature treatment, whereas *LHY* was downregulated. These four components, which are related to the regulation of the low-temperature stress response, play significant roles in the heat stress-responsive transcriptome. Recently, a targeted study also suggested a role for the evening-expressed clock components *TOC1* and *PRR5* in gating the molecular responses of select genes to warm temperature or high ambient temperature [60].

### 4.2. Clock Genes Involved in Thermomorphogenesis

The strategy of adaptation to ambient temperature stress allows plants to maintain biological activity at an optimal level even under fluctuating temperature conditions [39]. When *A. thaliana* is exposed to high temperatures during its growth period, there are increases in hypocotyl elongation and petiole extension; moreover, it forms small, thin leaves. These characteristics are known as thermomorphogenesis, and the plant moves the apical meristem away from the hot soil surface to promote evaporative cooling of leaves [68]. Thermomorphogenic adaptation responses help plants to efficiently manage body heat dissipation and leaf cooling, thereby optimizing growth and fitness in warm climates [69,70]. Thermomorphogenesis is mainly mediated by PHYTOCHROME INTERACTING FACTOR4 (PIF4), a basic helix-loop-helix transcription factor that promotes stem elongation [71]. PIF4 is a central growth regulator that directly activates several auxin biosynthesis genes, including *YUCCA 8* (*YUC8*), to increase the endogenous auxin level; this increase promotes hypocotyl elongation and hyponastic leaf growth [72,73]. PIF4 is also controlled by diverse environmental signals and endogenous programs. Warm temperatures increase *PIF4* expression, and the *pif4*-null mutant is defective in the growth response to warm temperature [74,75]. Furthermore, *PIF4* RNA expression is controlled by the circadian clock [76,77].

**Figure 2:**
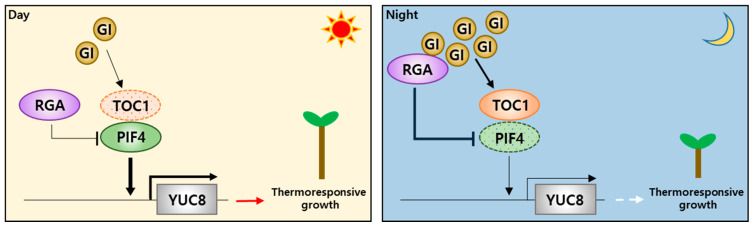
Schematic model for circadian gating of thermomorphogenesis. During the day, warm temperatures activate PHYTOCHROME INTERACTING FACTOR 4 (PIF4), which then activates auxin biosynthesis genes including *YUCCA8* (*YUC8*), promoting hypocotyl growth. However, in the evening and early night, TIMING OF CAB EXPRESSION 1 (TOC1) accumulates at high levels and directly inhibits PIF4, suppressing thermomorphogenesis [60]. Additionally, warm temperatures enhance the accumulation of the chaperone GIGANTEA (GI), which thermostabilizes the DELLA protein REPRESSOR OF GA1-3 (RGA). RGA stabilized by GI negatively regulates PIF4 protein during thermomorphogenesis [61].

Both TOC1 and PRR5, two evening element clock proteins, directly interact with PIF4; they inhibit PIF4-mediated growth promotion by inhibiting its transcriptional activity (Figure 2) [60,78]. The TOC1–PIF4 interaction can repress PIF4 activity without altering the ability of PIF4 to bind to the *YUC8*, *IAA19*, and *IAA29* promoters. TOC1 directly represses PIF4’s ability to activate target gene expression. Because PIF4 is required for thermomorphogenesis, including hypocotyl growth responses to warm temperature, it is possible that the TOC1–PIF4 interaction suppresses thermoresponsive growth. In an experimental analysis, the hypocotyls of *toc1-2* mutants were more elongated by transient high-temperature exposure compared with hypocotyls of the wild type, and *TOC1*-overexpressed (*TOC1*-OX) plants were completely insensitive to high-temperature treatment. *PRR5*-OX plants, but not other *PRR* family members (*PRR3*, *PRR7,* or *PRR9*), were also insensitive to warm temperature. Consistent with the thermo-insensitive hypocotyl growth of *TOC1*-OX, the warm-temperature activation of PIF4 target genes *YUC8*, *IAA19*, and *IAA29* was abolished in *TOC1*-OX. However, the expression of the early high temperature-induced gene *HEAT SHOCK PROTEIN 70* (*HSP70*) was induced at normal levels by warm temperatures in *TOC1*-OX. TOC1 and PRR5 inhibit the warm-temperature activation of PIF4 target genes through direct repression of PIF4 protein activity. Plant architectural adaptation to warm temperature, mediated by PIF4, enhances evaporative leaf cooling and thus may improve plant survival under heat stress [69,75]. This adaptive effect was considerably reduced in the *pif4* mutant, whereas *PIF4*-OX plants were more tolerant to heat stress than the wild type. Consistent with the TOC1 inhibition of PIF4 activity, *TOC1*-OX plants were less thermotolerant, but the *toc1prr5* double mutant showed more thermotolerance than wild-type plants when grown under warm temperatures. When grown under normal temperatures, the *TOC1*-OX and *toc1prr5* plants showed similar sensitivity to heat stress. These results indicate that adaptive thermotolerance requires PIF4 activation and a low level of TOC1, suggesting that PIF4 activation by daytime warm temperatures, allowed by troughs in the level of TOC1, enhances plant survival during heat stress.

Warm temperatures enhance the accumulation of the chaperone GIGANTEA (GI), which thermostabilizes the DELLA protein REPRESSOR OF GA1-3 (RGA) under long-day conditions, thereby attenuating PIF4-mediated thermomorphogenesis. Mutant studies provided insights concerning the role of GI in various abiotic stresses, as well as various growth and developmental processes. GI interacts with multiple PIF proteins that mediate diverse facets of light-signaling events and accompanying photomorphogenic responses in plants [79]. GI attenuates PIF4 function by stabilizing DELLA proteins, which function as negative regulators of PIF proteins [80], during thermomorphogenesis. The chaperone activity of GI protects substrate proteins, such as ZTL and RGA, from the ubiquitin–proteasome pathway [81]. HSP90 is a representative molecular chaperone that stabilizes a broad spectrum of cellular proteins in plants under high-temperature stress [82]. GI functions along with HSP90 during the maturation of ZTL proteins [83]. In an experimental analysis, HSP90 and GI accumulation under warm temperatures led to the intrinsic chaperone activity of GI, supporting the thermostabilization of the RGA protein during hypocotyl thermomorphogenesis [84]. These results indicate that the GI-mediated integration of photoperiodic and temperature information shapes thermomorphogenic rhythms, which enable plants to adapt to fluctuations in day length and temperature during seasonal transitions (Figure 2).

### 4.3. Circadian Clock Genes Involved in Heat Resistance

ZTL increases thermotolerance through a protein expression system [10]. In contrast to thermomorphogenesis, ZTL appears to regulate different high-temperature responses through distinct mechanisms; the thermotolerance defect in *ztl* mutants is not restored by *TOC1* and *PRR5* mutations [85]. Increased *ZTL* expression at high temperatures may promote plant survival under high-temperature stress. ZTL, an HSP90 client protein, improves heat resistance by resolving high-temperature-induced protein aggregation through polyubiquitination-mediated protein degradation [10]. HSP90 positively controls thermomorphogenesis by stabilizing the auxin receptor TRANSFORT INHIBITOR RESPONSE 1 (TIR1) [84]. HSP90 is associated with GI and regulates the maturation of ZTL, which is required for the nighttime degradation of TOC1 and PRR5. Overall, HSP90 regulates thermomorphogenesis through the ZTL-TOC1/PRR5 signaling module and the TIR1 auxin receptor. The *ztl-105* mutant exhibited reduced thermotolerance, accompanied by a decline in polyubiquitination and an increase in protein aggregate formation. ZTL and its interacting partner HSP90 were cofractionated with insoluble aggregates under heat stress, indicating that ZTL contributes to the thermoresponsive protein quality control machinery. Thus, ZTL-mediated protein quality control contributes to the thermal stability of the circadian clock.

## 5. Conclusions

In nature, plants frequently encounter temperature changes within various temperature regimes, and they are equipped with defense mechanisms to adapt to temperature stimuli. In the model plant *A. thaliana*, which has an optimal growth temperature of 22 °C, normal growth is possible in the range of 16–28 °C. Temperatures below 16 °C or above 29 °C are perceived as stress, leading to severely reduced growth, distorted plant structure and morphology, and decreased seed production [86]. Circadian clock genes play essential roles in allowing plants to maintain homeostasis through the accommodation of temperature changes in the normal temperature range or alterations of protein properties and morphogenesis at the cellular level. The molecular mechanism that buffers the clock in response to changes in cellular metabolism has been explored in various plant species and is considered an essential function for maintaining plant growth, development, and survival under extreme conditions through the stabilization of the clock cycle [26,42,87]. The clock genes also directly influence key crop traits [88]. Additionally, considering that clock genes are involved in responses to various environmental stresses such as salt and drought, studies of clock genes and expression regulation technologies are important for efforts to improve plant characteristics. Various efforts have been made to mutate clock genes to improve various crop characteristics, including RNAi and overexpression, as well as recent gene-editing techniques (Table 1) [89,90]. In recent years, global warming has emerged as an important ecological problem for vegetation and crop agriculture worldwide [86]; thus, further analyses of the relationships between temperature stress response and plant growth and development are necessary to secure future plant resources and food security.

## Figures and Tables

**Table 1:** Roles of circadian clock genes in response to environmental stress in different plant species.

Stress Conditions	Plant Species	Responsible Genes	References
Cold	*Oryza sativa*	*LUX*	Huang et al., 2023 [23]
	*Hordeum vulgare*	*CCA1*, *GI*, *PRR59*, *PRR73*, *PRR95*, *LUX*	Ford et al., 2016 [24]
	*Castanea sativa*	*TOC1*, *LHY*, *PRR5*, *PRR7*, *PRR9*	Ibañez et al., 2008 [25]
	*Brassica oleracea*	*CCA1*	Song et al., 2018 [26]
Drought	*Glycine max*	*LCL1*, *GmELF4*, *PRR-like*, *TOC1-like*, *LUX-like*, *PRR7-like* genes	Marcolino-Gomes et al., 2014 [27]
	*Zea mays*	*CCA1*	Tian et al., 2019 [28]
Salt	*Brassica rapa*	*GI*	Kim et al., 2016 [29]
	*G. max*	*E2* (an ortholog of *GI*)	Dong et al., 2022 [30]
	*G. max*	*J* (an ortholog of *ELF3*)	Cheng et al., 2020 [31]
	*O. sativa*	*GI*, *EC1*	Wang et al., 2021 [32]
	*O. sativa*	*PRR73*	Wei et al., 2020 [33]
Osmotic	*H. vulgare*	*ELF3*	Habte et al., 2014 [34]
Cold, drought, heat, salt	*Ipomoea batatas*	*GI*	Tang et al., 2017 [35]
Salinity, osmotic, drought	*O. sativa*	*CCA1*	Wei et al., 2022 [36]
Salt, cold	*B. rapa*	*GI*	Xie et al., 2015 [37]
